# Efficacy and safety of remimazolam tosilate versus propofol in patients undergoing day surgery: a prospective randomized controlled trial

**DOI:** 10.1186/s12871-023-02092-2

**Published:** 2023-05-26

**Authors:** Wenchen Luo, Minli Sun, Jie Wan, Zhenyu Zhang, Jian Huang, Jinlin Zhang, Wanxia Xiong, Lirong Xia, Peiyao Xu, Changhong Miao, Xuesong Zhang, Mingyue Liu, Jing Zhong

**Affiliations:** 1 Department of Anesthesiology, Zhongshan Hospital Fudan University, Shanghai, 200032 People’s Republic of China; 2 Fudan Zhangjiang Institute, Shanghai, People’s Republic of China; 3 Department of Anesthesiology, Zhongshan Wusong Hospital Affiliated to Fudan University, Shanghai, People’s Republic of China; 4 School of Anesthesiology, Xuzhou Medical University, Xuzhou, People’s Republic of China; 5 Jiangsu Jiangyin People’s Hospital, Jiangyin, Jiangsu People’s Republic of China; 6 Shanghai Key Laboratory of Perioperative Stress and Protection, Shanghai, People’s Republic of China

**Keywords:** Remimazolam tosilate, Propofol, Flumazenil, General anesthesia, Day surgery

## Abstract

**Background:**

Remimazolam tosilate (RT) is a novel short-acting GABA (A) receptor agonist that has a rapid recovery from procedural sedation and can be fully reversed by flumazenil. To date, there have been relatively few articles comparing RT and propofol for general anesthesia. This study aimed to assess the efficacy and safety of RT with or without flumazenil compared with propofol in general anesthesia for day surgery.

**Methods:**

115 patients scheduled for day surgery were randomized into three groups: RT (n = 39), RT + flumazenil (n = 38) and propofol (n = 38). The primary endpoints were anesthesia induction time and time until fully alert. Anesthesia success rate, bispectral index (BIS) values, injection pain, opioid and vasopressor dosages, postoperative recovery profiles and perioperative inflammatory and cognitive changes were assessed. Any adverse events were recorded.

**Results:**

Induction times were similar among the three groups (P = 0.437), but the median time until fully alert in patients treated with RT was longer than that of the propofol or RT + flumazenil groups (17.6 min vs. 12.3 min vs. 12.3 min, P < 0.001). The three groups had comparable postoperative recovery quality and inflammatory and cognitive state changes (P > 0.05). Smaller percentages of patients who received RT (26.3%) and RT + flumazenil (31.6%) developed hypotension during anesthesia maintenance compared with propofol (68.4%), and consequently less ephedrine (P < 0.001) and phenylephrine (P = 0.015) were needed in the RT group. Furthermore, serum triglyceride levels were lower (P < 0.001) and injection pain was much less frequent in the RT with or without flumazenil groups compared with the propofol group (5.3% vs. 0% vs. 18.4%).

**Conclusion:**

RT permits rapid induction and comparable recovery profile compared with propofol in general anesthesia for day surgery, but has a prolonged recovery time without flumazenil. The safety profile of RT was superior to propofol in terms of hypotension and injection pain.

**Trial registration:**

The study was registered at Chinese Clinical Trial Registry http://www.chictr.org.cn/ (Registration date: 19/7/2021; Trial ID: ChiCTR2100048904).

**Supplementary Information:**

The online version contains supplementary material available at 10.1186/s12871-023-02092-2.

## Background

Day surgery under general anesthesia has been associated with a shortened hospital stay and earlier mobilization [1]. All commonly-used intravenous (IV) anesthetics currently, including propofol and midazolam, have their respective advantages and disadvantages. Propofol is the most frequently used IV anesthetic for day surgery because of its excellent sedative properties and short terminal half-life. It also reduces the generation of pro-inflammatory cytokines and has been shown to exert a neuroprotective effect by maintaining Th17/Treg cell balance [2]. Despite being widely used, propofol has several disadvantages, including injection pain, cardiorespiratory depression, metabolic acidosis and hyperlipidemia [3]. Midazolam, the most commonly used benzodiazepine, causes less circulatory depression than propofol but has a comparatively longer induction and recovery time due to its longer half-life and active metabolite [4, 5]. Remimazolam besylate, an ultra-short-acting benzodiazepine agonist with propofol’s fast on- and offset characteristics [4], has been shown to be safe and effective in the induction and maintenance of general anesthesia [6, 7]. However, a previous study reported that remimazolam besylate for general anesthesia had prolonged mean time from the end of study drug to eye opening compared with propofol (14.9 ± 11.1 min vs. 10.3 ± 5.1 min) [7]. A temporary reduction in recovery quality compared with propofol was also reported in patients who underwent urologic surgery using remimazolam besylate [8].

Similar to remimazolam besylate in structure and pharmacological properties [9, 10], remimazolam tosilate (RT) is a new short-acting GABA (A) receptor agonist with a tosilate counterion. Like remimazolam besylate, RT has a short half-life which results in the quick acting onset and recovery than currently available short-acting sedatives [9]. In addition, compared with the same dose level of remimazolam besylate, a slightly different recovery time was observed in the dose range of 0.1–0.35 mg/kg of RT, which was equivalent to remimazolam besylate of 0.075-0.3 mg/kg after labeling dose conversion, with a median time to fully alert ranging from 0 to 21.5 min for RT in comparison with 5.5–31.5 min for remimazolam besylate [9]. Recent study established a non-inferior sedation success rate with RT compared with propofol for upper gastrointestinal endoscopy [11] and colonoscopy [12]. RT permits rapid recovery from procedural sedation [12] and is less likely to cause cardiovascular and respiratory depression than propofol [11, 12]. However, little is known about the utility of RT in general anesthesia. Based on the prolonged recovery from general anesthesia of remimazolam besylate and given the complete antagonistic action of flumazenil, a benzodiazepine receptor antagonist, the efficacy and safety of RT in conjunction with flumazenil was taken into account.

We designed this study to assess the efficacy and safety of RT with or without flumazenil compared with propofol in general anesthesia for day surgery with particular interest in postoperative recovery quality and inflammatory and cognitive changes.

## Methods

### Ethical approval

This study was a single-center randomized, single-blinded, positive-controlled, parallel trial performed in Zhongshan Hospital Fudan University in Shanghai. It was approved by the Ethics Committee of Zhongshan Hospital (Number B2021–360) and was registered with the Chinese Clinical Trial Registry prior to patient enrollment (ChiCTR2100048904; Principal investigator: CM; Date of registration: July 19, 2021). The trial was modified on September 23, 2021 to add an additional arm to test RT + flumazenil. Patients were recruited from October 2021 to December 2021. Written informed consent was obtained from all participants included in the study. This manuscript adheres to the applicable Consolidated Standards of Reporting Trials guidelines.

### Participants

This trial included male and female patients aged 18 to 75 years old who had an American Society of Anesthesiologists physical status (ASA) of I or II, a body mass index of 18 to < 30 kg/m^2^ and underwent day surgery utilizing general anesthesia with a laryngeal mask airway (LMA). Participants were excluded if they had a history of a benzodiazepine allergy, a history of benzodiazepine or opioid use within 1 month of surgery, a history of alcohol abuse, clinically significant renal or hepatic dysfunction, significant cardiorespiratory instability (heart failure, acute lung injury, hypovolemia or sepsis), participated in different drug trials within 3 months of enrollment or were pregnant or breast-feeding.

### Randomization and blinding

Participants were divided into three groups in a 1:1:1 ratio via block randomization: RT, RT + flumazenil and propofol. The randomization chart was generated using a web-based randomization system (http://www.randomization.com, USA) that employed a Wichmann and Hill number generator as modified by McLeod. Sealed and numbered envelopes were handed to an anesthesiologist who was involved in drug administration. Intraoperative data was recorded by an anesthesiology assistant. A blinded investigator who was not directly involved in intraoperative anesthesia collected all postoperative data. Both patients and surgeons were blinded to group allocation. Because propofol and RT had different colors and infusion methods, the anesthesiologists and anesthesiology assistants were unblinded to group allocation.

### Interventions

General anesthesia was induced with RT (0.3 mg/kg, iv) (Jiangsu Hengrui Medicine Co, Ltd, Jiangsu, China) or propofol (2.0–2.5 mg/kg, iv) (AstraZeneca, United Kingdom) in combination with sufentanil (0.2–0.4 µg/kg, iv). If loss of consciousness (LoC) did not occur within 3 min, an IV dose of RT (0.1 mg/kg) or propofol (1.0 mg/kg) was administered. After LoC was confirmed, muscular paralysis was achieved using rocuronium (0.2–0.4 mg/kg, iv) and an LMA was inserted.

RT was maintained at 1–3 mg/kg/h and propofol was maintained within a range of 6–12 mg/kg/h. Remifentanil was administered at an infusion rate of 0.05–0.15 µg/kg/min. Bispectral index (BIS) was used to evaluate anesthesia depth. IV anesthetic infusion was discontinued when the final surgical dressing was applied. The participant who developed hypotension, defined as systolic blood pressure (SBP) < 80% of baseline, was administered a bolus dose of 6 mg ephedrine or 0.1 mg phenylephrine that was repeated as necessary. Sinus bradycardia, defined as a heart rate (HR) below 40 beats/min, was treated with 0.5 mg of atropine.

Towards the end of the surgery, nalmefene (20 µg, iv), neostigmine (0.04 mg/kg, iv) and atropine (0.02 mg/kg, iv) were allowed to be administered as necessary. In the RT + flumazenil group, 0.2 mg flumazenil (Zhejiang Aotuokang Medicine Co, Ltd, Zhejiang, China) was administered 10 min after the discontinuation of RT. If necessary, a repeated dose of 0.1 mg flumazenil was permitted every minute until the total dose reached 1 mg. Blood pressure, HR, blood oxygen saturation (SpO_2_) and the BIS were monitored and recorded throughout the course of anesthesia.

### Outcome variables

The primary outcomes were anesthesia induction time and time until the patient was fully alert post-operatively. Induction time was defined as the time from when the participant became unresponsive to painful stimulation (namely the squeezing of his/her trapezius) after the start of anesthesia administration. Time to fully alert was defined as the time from the stopping of anesthetic dosing to the patient accurately stating his/her date of birth [13].

Key secondary endpoints included: anesthesia success rate, defined as the absence of (1) intraoperative awakening or recall, (2) the need for rescue sedative medication and (3) body movement; LMA insertion time; the BIS values; the number of patients with BIS values > 60 or < 40 during anesthesia maintenance; % time BIS > 60 during anesthesia maintenance, defined as the percentage of time with BIS values > 60 in the whole anesthesia maintenance period; the recovery time including the time to eye opening, time to LMA extraction and time to the third consecutive Aldrete score ≥ 9 in the post-anesthesia care unit (PACU); postoperative delirium. Time to the third consecutive Aldrete score ≥ 9 was defined as the period from LMA extraction to the measurement of the third consecutive Aldrete score ≥ 9. Postoperative delirium was assessed prior to PACU discharge using the Diagnostic and Statistical Manual of Mental Disorders. After stopping the anesthetic, the dosages of opioids and vasoactive drugs were also recorded. Patients were under monitoring in PACU 60 min after flumazenil administration or 60 min after entering PACU to assess the recovery profiles, especially the sedation status after flumazenil administration. Recovery profiles were assessed based on the scores of the following assessments: modified observer’s alertness/sedation scale (MOAA/S) before the surgery and 60 min after flumazenil administration or 60 min after entering PACU; Aldrete score 60 min after entering PACU; mini-mental state examination (MMSE) before the surgery, in PACU and on 1 day after surgery (POD1); visual analogue scale (VAS) before the surgery, in PACU and on POD1; the post-operative quality of recovery scale (PostopQRS) before the surgery, on POD1 and 14 days after surgery (POD14); a telephone interview to evaluate cognitive status on POD14. Residual cognitive effects (more than 7 days) following ambulatory anesthesia in middle-aged patients have been previously reported [14]. The postoperative follow-up time point was therefore extended to POD14 to permit a comprehensive evaluation of cognitive recovery and complications [15, 16]. Vital signs including SpO_2_, HR, SBP and diastolic blood pressure (DBP) were recorded at twelve time points: before induction (T1), after induction (T2), after LMA insertion (T3), 5 min after the beginning of surgery (T4), 10 min after the beginning of surgery (T5), 10 min before stopping the study drug (T6), 5 min before stopping the study drug (T7), immediately after stopping the study drug (T8), 10 min after stopping the study drug (T9), 15 min after stopping the study drug (T10), LMA extraction (T11) and before leaving the operating room (T12).

Blood samples were drawn before anesthesia induction and at PACU discharge to measure inflammatory factors and biomarkers relevant to cognitive and lipid profiles, such as neuropeptide Y (NPY), interleukin (IL)-8, IL-1β, IL-10, triglyceride (TG) and very low density lipoprotein (VLDL) levels. The above factors were measured using enzyme-linked immunosorbent assay kits obtained from XLPCC (Shanghai, China) according to the manufacturer’s instructions. In order to explore whether RT had the similar effect to propofol in terms of inflammatory factors activation and Th17/Treg cell balance [2], neutrophil (CD11b/CD18) and Treg cell (CD39/CD73) surface markers were also measured in peripheral blood samples according to the manufacturer’s instructions (BD, San Diego, CA, USA) and then detected using flow cytometry.

Drug-related adverse events (AEs) including hypotension, sinus bradycardia and hypoxia (defined as SpO_2_ < 90%) were monitored throughout this study. Any AEs related to flumazenil were also recorded. In addition, the incidence of injection pain, intraoperative awareness, body movement, airway intervention after LMA extraction, postoperative nausea and vomiting and complications up to POD14 were calculated.

### Statistical analysis

The sample size required for this study was based on pre-trial estimates of induction time and the time until fully alert. The mean induction times of the RT, RT + flumazenil and propofol groups were 55s, 58s and 51s, respectively, and the standard deviations (SD) for each group were 5s, 3s and 2s, respectively. Assuming an α = 0.05 and a power of 80% (two-sided tests), we calculated that a sample of 33 participants for each arm were required using the PASS 15 software (NCSS Corp., USA). The same method was used to estimate that at least 15 participants per arm were required to evaluate time until fully alert. Allowing for dropouts and non-evaluable data, a minimum of 38 participants were recruited for each group.

Analysis was performed according to the modified intention-to-treat principle. Data were presented using mean ± SD or median (25th, 75th percentiles) for continuous variables, and frequency counts and percentages for nominal variables. The Shapiro–Wilk test was used to determine whether continuous outcomes were normally distributed. Normally distributed data were tested using a one-way analysis of variance (ANOVA), with pairwise comparisons made with the least square mean values t test. Nonparametric data were tested with the Kruskal–Wallis test, with pairwise comparisons made with the Wilcoxon-rank sum test. Categorical outcomes were compared with the χ2 analysis or Fisher’s exact test in the setting of low expected cell counts. Endpoints assessed at different times were analyzed using the mixed-model repeated measures analysis. All outcomes were considered exploratory in nature and thus no correction for multiple comparisons was made. All statistical tests were two-tailed, and significance was defined using a P value < 0.05. All statistical analyses were performed using SPSS19.0 software (IBM, Armonk, NY, USA) and R software version 4.1.0 (The R Foundation, Vienna, Austria).

## Results

### Patient demographic and clinical characteristics

**Fig. 1:**
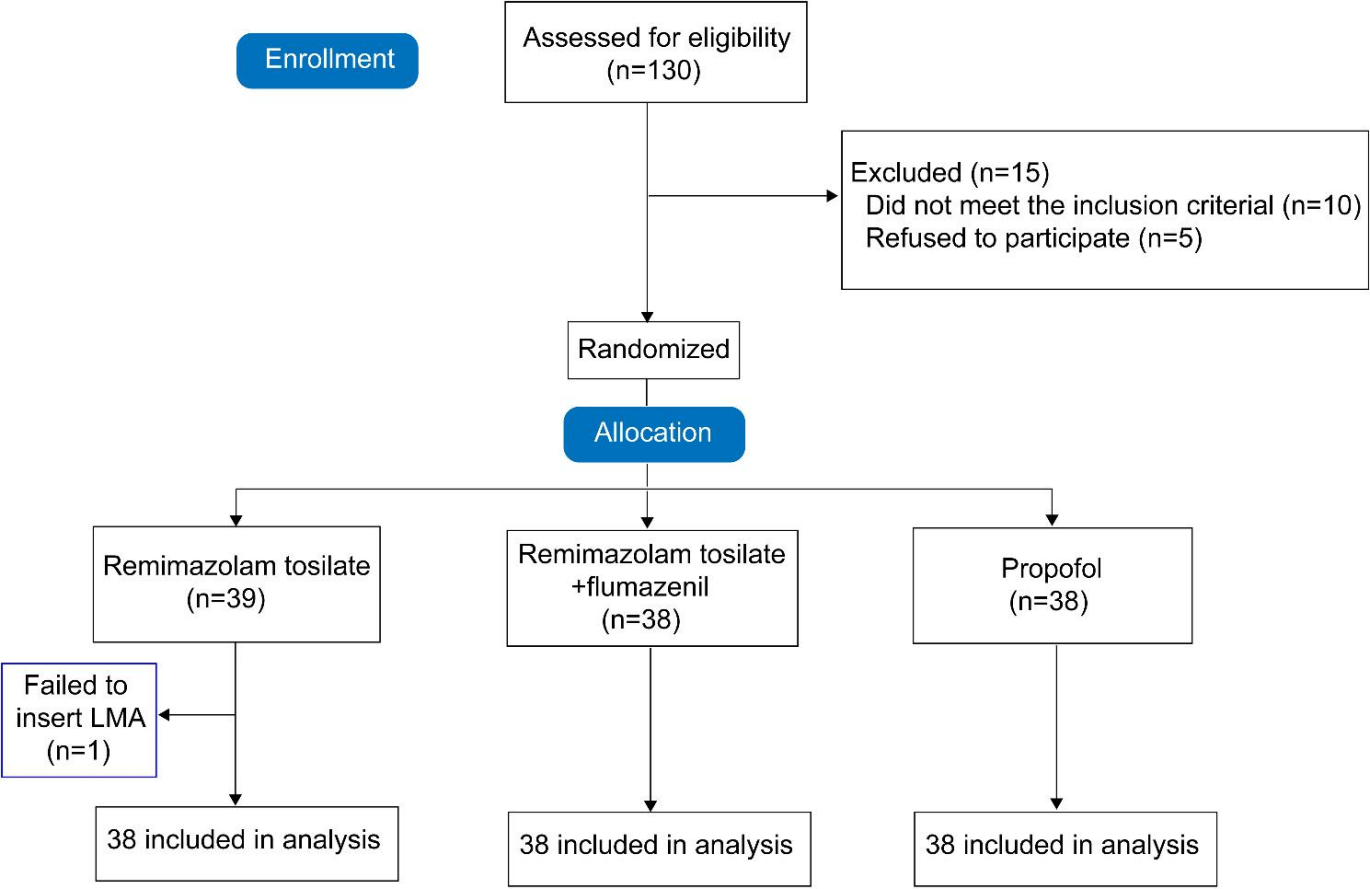
Participant flow of the study. LMA, laryngeal mask airway

A total of 130 participants were screened for eligibility. Fifteen were excluded, leaving 115 patients to be randomized into three groups: 39 to the RT group, 38 to the RT + flumazenil group and 38 to the propofol group (Fig. 1). One patient in the RT group did not receive the intervention or follow-up due to failed LMA insertion. Patient and surgery characteristics are shown in Table 1. There were no relevant imbalances between the groups at baseline including surgery type and preoperative laboratory tests such as inflammatory biomarkers neutrophil/lymphocyte ratio and platelet/lymphocyte ratio (P > 0.05; Table 1).

**Table 1:** Baseline characteristics of study participants

Variables	RT (n = 38)	RT + flumazenil (n = 38)	Propofol (n = 38)	*P* value
Age (y)				
Mean ± SD	43.5 ± 15.6	44.7 ± 16.8	44.3 ± 18.1	0.951
≥ 65	6(15.8)	7(18.4)	7(18.4)	0.941
Male	14(36.8)	22(57.9)	19(50.0)	0.179
Weight (kg)	63.6 ± 12.6	66.6 ± 11.3	65.9 ± 13.5	0.540
Height (cm)	166.7 ± 8.6	168.7 ± 7.8	168.1 ± 9.1	0.568
BMI (kg/m^2^)	22.7 ± 3.4	23.3 ± 3.3	23.2 ± 3.7	0.729
ASA physical status				0.868
I	17(44.7)	17(44.7)	19(50.0)	
II	21(55.3)	21(55.3)	19(50.0)	
Comorbid medical conditions				
Hypertension	10(26.3)	10(26.3)	5(13.2)	0.278
Diabetes	5(13.2)	1(2.6)	2(5.3)	0.270
Coronary heart disease	1(2.6)	0(0.0)	1(2.6)	1.000
Thyroid disease	3(7.9)	1(2.6)	2(5.3)	0.870
Arrhythmia	4(10.5)	4(10.5)	4(10.5)	1.000
Surgery type				0.112
Urology	14(36.8)	24(63.2)	20(52.6)	
Obstetrics and Gynecology	16(42.1)	8(21.1)	7(18.4)	
General surgery	7(18.4)	6(15.8)	10(26.3)	
Thoracic surgery	1(2.6)	0(0.0)	1(2.6)	
Preoperative laboratory tests				
Neutrophil / lymphocyte ratio, %	2.07(1.57–2.68)	2.26(1.81–3.52)	2.28(1.76-3)	0.212
Platelet / lymphocyte ratio, %	115.47(106.40-147.97)	133.93(109.89-165.58)	138.99(105.92-164.67)	0.371
ALT (U/L)	13.50 (9.00-20.25)	14.00 (12.00-18.25)	15.00 (10.00-22.75)	0.663
Urea (mmol/L)	4.80 (4.20–6.25)	5.35 (4.38–6.62)	4.80 (4.23–5.38)	0.269
TBIL (µmol/L)	8.55 (5.78–12.28)	10.80 (8.90–12.80)	10.05 (7.62–12.47)	0.222
Duration of surgery (min)	42.2 ± 27.7	38.6 ± 29.1	38.7 ± 25.1	0.767

Data are presented as mean ± SD, numbers (percentages) or median [IQR], as appropriate

Abbreviations: ASA, American Society of Anesthesiologists; BMI, body mass index; RT, remimazolam tosilate; SD, standard deviation; ALT, alanine aminotransferase; TBIL, total bilirubin

### Primary outcomes

There was no significant difference in anesthesia induction time among the three groups (P = 0.437; Table 2). However, a significant increase in the time to fully alert was detected in the RT group compared with the propofol (P < 0.001) and RT + flumazenil groups (P < 0.001; Table 2). Time to fully alert was comparable between the propofol and RT + flumazenil groups (P = 0.949; Table 2).

**Table 2:** Study outcomes

Perioperative Details		RT (Arm A, n = 38)	RT + flumazenil (Arm B, n = 38)	Propofol (Arm C, n = 38)	*P* value	Arm A vs. Arm C	Arm B vs. Arm C	Arm A vs. Arm B
**Primary endpoint**								
Induction time (s)		50.0(45.0-55.8)	49.5(43.8–60.0)	51.0(41.0-60.3)	0.437			
Time to fully alert (min)		17.6(12.9–24.1)	12.3(11.6–13.0)	12.3(9.4–16.4)	< 0.001	< 0.001	0.949	< 0.001
**Secondary endpoints**								
Success rate of anesthesia		37(97.4)	37(97.4)	37(97.4)	1.000			
LMA insertion time (s)		129.0(120.0-139.0)	125.0(119.0-140.0)	140.0(118.5–155.0)	0.820			
Time to eye opening (min)		16.8(11.4–22.4)	11.3(11.1–12.2)	10.2(7.8–15.5)	< 0.001	< 0.001	0.570	0.001
Time to LMA extraction (min)		17.2(12.2–22.8)	11.7(11.3–12.5)	11.2(8.2–16.1)	< 0.001	< 0.001	0.712	0.001
Time to the third consecutive Aldrete score ≥ 9 (min)		49.0(43.5–52.0)	33.5(28.0-37.3)	33.0(30.0-35.8)	< 0.001	< 0.001	0.664	< 0.001
Delirium at PACU		0	0	0	1.000			
BIS								
Baseline		89.0(86.0–94.0)	93.0(89.5–95.0)	94.0(88.0–96.0)	0.216			
LoC		70.0(62.0–74.0)	66.5(62.8–76.0)	55.5(48.0-66.3)	0.002	0.002	0.002	0.969
LMA insertion		63.5(58.0-71.3)	67.0(62.3–74.0)	51.0(43.0–60.0)	< 0.001	< 0.001	< 0.001	0.212
LMA extraction		86.0(82.3–87.0)	87.0(84.0-89.3)	87.0(87.0–90.0)	0.009	0.002	0.246	0.078
Exiting operating room		86.0(83.3–87.8)	87(84.0–91.0)	87.0(85.5–90.5)	0.034	0.005	0.618	0.050
N BIS values during anesthesia maintenance								
BIS > 60		37(97.4)	35(92.1)	16(42.1)	< 0.001	< 0.001	< 0.001	0.615
BIS<40		3(7.9)	2(5.3)	11(28.9)	0.005	0.018	0.006	1.000
% time BIS > 60 during anesthesia maintenance (%)		80.0 (53.4–89.7)	73.6 (50.0-90.5)	0.0 (0.0-39.7)	< 0.001	< 0.001	< 0.001	0.954
RT dose (mg)		49.2(22.5–69.0)	36.0(28.0-75.2)		0.941			
Sufentanil (µg)		14.8(11.9–20.0)	15.0(12.6–19.5)	14.9(13.0–20.0)	0.805			
Remifentanil (µg)	250.0(121.45–340.0)		140.4(98.0-320.0)	140.0(100.0-261.0)	0.301			
Vasopressors		12(31.6)	12(31.6)	27(71.1)	< 0.001	0.001	0.001	1.000
Ephedrine (mg)		0.0(0.0–0.0)	0.0(0.0–0.0)	6.0(0.0–6.0)	< 0.001	0.001	< 0.001	0.847
Phenylephrine (mg)		0.0(0.0–0.0)	0.0(0.0–0.0)	0.0(0.0-0.1)	0.048	0.015	0.209	0.219

Data are presented as median (interquartile range) or numbers (percentages), as appropriate

Abbreviations: BIS, bispectral index; IQR, interquartile range; LMA, laryngeal mask airway; LoC, loss of consciousness; N BIS values, number of patients with corresponding BIS values; PACU, postanesthesia care unit; RT, remimazolam tosilate; % time BIS > 60 during anesthesia maintenance, the percentage of time with BIS values > 60 in the whole anesthesia maintenance period

### Secondary outcomes

**Fig. 2:**
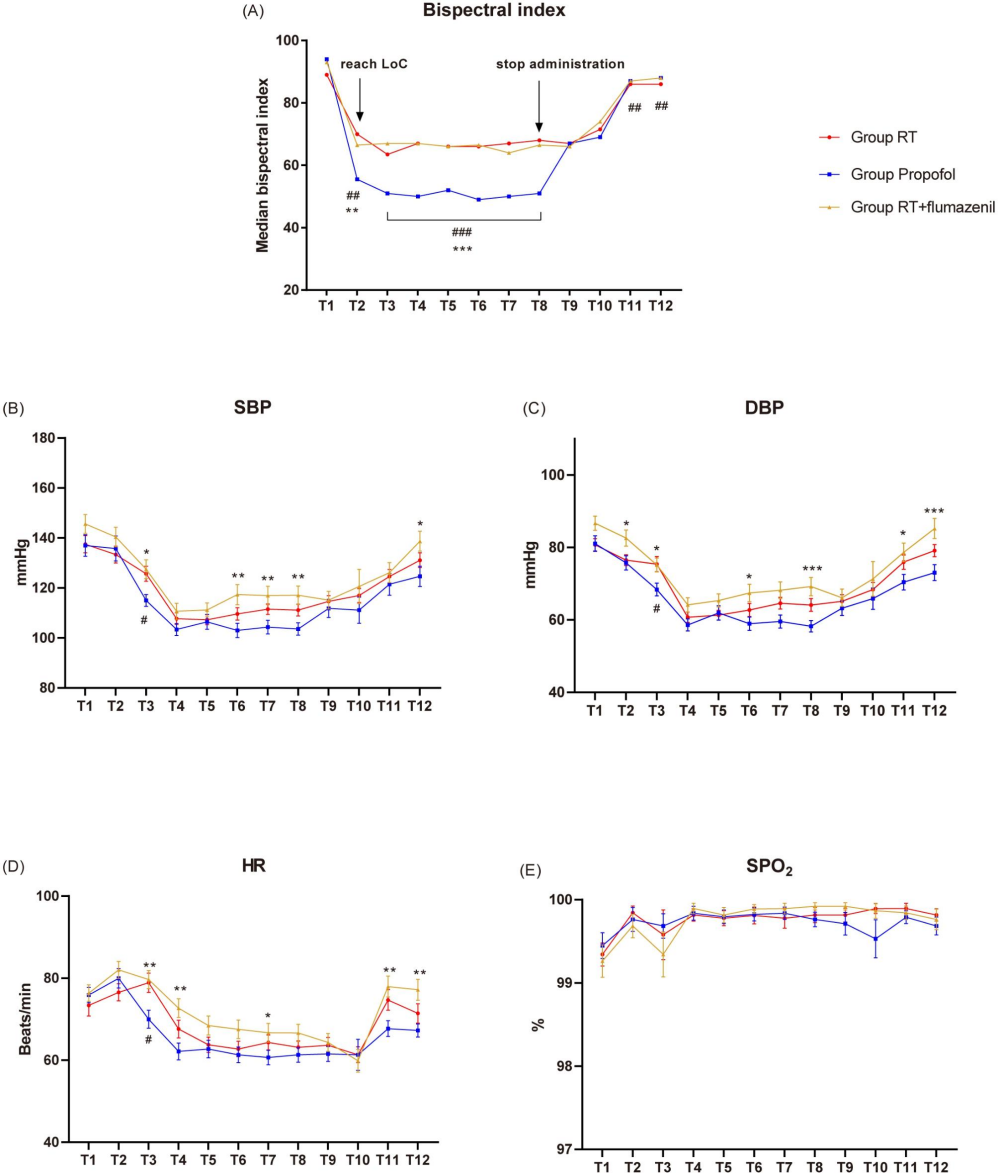
Vital signs and bispectral index during the entire procedure. Evaluation of the levels of (**A**) bispectral index, (**B**) SBP, (**C**) DBP, (**D**) HR and (**E**) SpO_2_ before induction (T1), after induction (T2), after laryngeal mask airway insertion (T3), 5 min after the beginning of surgery (T4), 10 min after the beginning of surgery (T5), 10 min before stopping the study drug (T6), 5 min before stopping the study drug (T7), immediately after stopping the study drug (T8), 10 min after stopping the study drug (T9), 15 min after stopping the study drug (T10), after laryngeal mask airway extraction (T11) and before leaving the operating room (T12). Data are shown as mean ± SE or median. SBP, systolic blood pressure; DBP, diastolic blood pressure; HR, heart rate; SpO_2_, blood oxygen saturation; RT, remimazolam tosilate; LoC, loss of consciousness. ^*^P < 0.05, ^**^P < 0.01, ^***^P < 0.001, RT + flumazenil versus propofol group; ^#^P < 0.05, ^##^P < 0.01, ^###^P < 0.001, RT versus propofol group

Sedation success rates were equivalent among the three groups, with 97.4% each (P = 1.000; Table 2). Time for LMA insertion was also similar among the three groups (P > 0.05 for all comparisons; Table 2). During induction, patients who received RT with or without flumazenil reached LoC with BIS values higher than propofol (RT vs. propofol, P = 0.002; RT + flumazenil vs. propofol, P = 0.002; Table 2; Fig. 2A). The same trend existed at T3-T8 time points (P < 0.001; Fig. 2A). However, BIS values were lower in the RT group compared to the propofol group at T11-T12 time points (P = 0.002, 0.005 respectively; Table 2; Fig. 2A). The number of patients with BIS values > 60 during anesthesia maintenance and % time BIS > 60 during anesthesia maintenance were significantly increased in the RT and RT + flumazenil groups compared with propofol group (P < 0.001 for all comparisons; Table 2). Conversely, fewer patients in the RT (P = 0.018) and RT + flumazenil groups (P = 0.006) had BIS values < 40 compared with the propofol group, indicating deeper sedation with propofol (Table 2). However, no patients in this study reported intraoperative awareness.

During the recovery phase, all of the patients in the RT + flumazenil group received flumazenil. The average dose of flumazenil was 0.27 mg and the maximum dose was 0.70 mg. No re-sedation occurred 60 min after flumazenil administration according to the MOAA/S scores (5.00 ± 0.00 for all three groups) and Aldrete scores (10.00 ± 0.00 for all three groups) in PACU. Time to eye opening, LMA extraction and the third consecutive Aldrete score ≥ 9 were statistically shorter in the propofol group and RT + flumazenil group compared with the RT group (P ≤ 0.001 for all comparisons; Table 2).

SpO_2_, HR, SBP and DBP were shown in Fig. 2. There were no significant differences in SpO_2_ among the three groups at T1–T12 (P > 0.05; Fig. 2E); further, baseline SBP, DBP and HR data were also comparable (P > 0.05; Fig. 2B-D). However, SBP was significantly higher at T3, T6-T8 and T12 (P < 0.05; Fig. 2B), DBP was significantly higher at T2, T3, T6, T8, T11 and T12 (P < 0.05; Fig. 2C), and HR was significantly higher at T3, T4, T7, T11 and T12 (P < 0.05; Fig. 2D) in the RT + flumazenil group compared with the propofol group. SBP, DBP and HR were all significantly higher in the RT group than those in the propofol group at T3 (P < 0.05; Fig. 2B-D). Fewer patients who received RT with or without flumazenil (31.6% and 31.6%) required vasopressors compared with propofol (71.1%, P < 0.001). Ephedrine (P < 0.001) dosage was significantly decreased in both RT groups compared with the propofol group, and phenylephrine (P = 0.015) dosage was significantly lower in the RT group than that in the propofol group (Table 2).

There was no postoperative delirium in any of the three groups prior to PACU discharge and on POD 1 and 14. There were no significant between-group differences in MMSE (PACU, P = 0.724; POD1, P = 0.117) or in PostopQRS scores (POD1, P = 0.072; POD14, P = 0.264) compared with baseline measurements (Table 3). Similarly, no differences were observed among the three groups in the telephone interview for cognitive status on POD14 (P = 0.500; Table 3). The VAS scores were comparable among the three groups with similar dosages of sufentanil (P > 0.05 for all comparisons; Table 2) both in PACU and on POD1 (PACU, P = 0.853; POD1, P = 0.356; Table 3). The serum levels of IL-8, IL-1β and IL-10 were similar before and after anesthesia among the three groups (P > 0.05 for all comparisons; Supplementary Fig. 1A–C). There were no significant differences in the expression of CD39(+), CD73(+), CD39(+)CD73(+), CD11b(+), CD18(+) and CD11b(+)CD18(+) among the three groups before and after anesthesia (P > 0.05 for all comparisons; Supplementary Table 1).

**Table 3:** Cognitive and recovery profile assessments

Group	RT (n = 38)	RT + flumazenil (n = 38)	Propofol (n = 38)	RT (n = 38)	RT + flumazenil (n = 38)	Propofol (n = 38)	RT vs. RT + flumazenil	RT vs. Propofol	RT + flumazenil vs. Propofol	
Time				Least Square Mean Change			Estimated Difference			*P value*
MMSE										
Preoperative	27.47 ± 3.24	27.71 ± 2.40	27.28 ± 2.20							
PACU	27.66 ± 3.08	27.68 ± 2.31	27.22 ± 2.77	0.18 ± 0.23	0.00 ± 0.24	-0.08 ± 0.24	0.18(-0.63-0.99)	0.26(-0.55-1.08)	0.08(-0.74-0.90)	0.724
POD1	28.45 ± 2.61	28.21 ± 1.91	27.56 ± 2.45	0.58 ± 0.16	0.24 ± 0.16	0.11 ± 0.17	0.34(-0.21-0.90)	0.47(-0.10-1.03)	0.13(-0.44-0.69)	0.117
VAS										
Preoperative	0.00 ± 0.00	0.11 ± 0.65	0.00 ± 0.00							
PACU	1.33 ± 1.55	1.44 ± 1.65	1.53 ± 1.94	1.33 ± 1.55	1.53 ± 1.94	1.33 ± 1.53	-0.20(-0.97-0.57)	-0.01(-0.80-0.77)	-0.18(-0.97-0.60)	0.853
POD1	0.63 ± 1.05	0.89 ± 1.10	1.03 ± 1.48	0.63 ± 1.05	1.03 ± 1.48	0.78 ± 1.35	-0.40(-0.95-0.16)	-0.28(-0.85-0.29)	-0.11(-0.68-0.46)	*0.356*
PostopQRS										
Preoperative	65.74 ± 3.47	66.05 ± 3.63	65.72 ± 3.17							
POD1	65.61 ± 3.76	64.39 ± 2.95	65.03 ± 3.42	-0.17 ± 0.43	-1.58 ± 0.43	-0.74 ± 0.44	1.41(-0.07-2.89)	0.57(-2.07-0.93)	-0.84(-2.34-0.66)	0.072
POD14	65.31 ± 2.76	65.30 ± 3.01	65.74 ± 2.74	-0.33 ± 0.30	-1.01 ± 0.30	-0.55 ± 0.32	0.68(-0.34-1.69)	0.22(-0.84-1.27)	-0.46(-1.51-0.59)	0.264
Telephone Interview for Cognitive Status										
POD14	33.16 ± 3.54	32.85 ± 3.86	32.00 ± 4.15							0.500

Data are presented as mean ± SD. The estimated difference is the least-squares mean ± SE change from baseline among the three groups at the corresponding time respectively

Abbreviations: CI, confidence interval; MOAA/S, Modified Observer’s Alertness/Sedation scale; MMSE, mini-mental state examination; PACU, postanesthesia care unit; POD1, 1 day after surgery; POD14, 14 days after surgery; VAS, visual analogue scale; PostopQRS, post-operative quality of recovery scale; RT, remimazolam tosilate; SD, standard deviation; SE, standard error

With respect to lipid metabolism, extensive changes in TG were observed in the propofol group in the PACU. The strongest increase of TG after anesthesia was observed in the propofol group compared with the RT with or without flumazenil groups (1.88 ± 1.28 mmol/L, 1.33 ± 0.70 mmol/L, 1.43 ± 0.75 mmol/L, respectively; P < 0.001; Supplementary Fig. 1E). The baseline VLDL measurement in propofol group was significantly lower than that of both RT groups (P < 0.001; Supplementary Fig. 1F). However, following covariance analysis PACU VLDL levels were comparable among the three groups (P = 0.728; Supplementary Fig. 1F). Changes in NPY, which was found to have hypnotic properties and be increased during the sleep inhibition phase [17], were not significant among the three groups (P > 0.05 for all comparisons; Supplementary Fig. 1D).

### Adverse events

No patients had a serious AE. The distributions of AEs pertaining to hemodynamics and other complications are shown in Table 4. One patient (2.6%) in the propofol group developed hypoxemia and required an airway intervention in the recovery phase. Postoperative nausea and vomiting occurred in two patients (5.3%) treated with propofol. Smaller percentages of patients who received RT (26.3%) and RT + flumazenil (31.6%) became hypotensive during anesthesia maintenance compared with those who received propofol (68.4%). The incidence of injection pain was 18.4% in the propofol group, 0% in RT group and 5.3% in RT + flumazenil group. No AEs related to flumazenil were observed. No intraoperative arousal or recall was reported in any group. No complications were reported during the POD14 telephone interview in any group.

**Table 4:** Drug-related adverse events

Adverse events	RT (n = 38)	RT + flumazenil (n = 38)	Propofol (n = 38)
Hypotension			
Anesthesia induction	3(7.9)	9(23.7)	4(10.5)
Anesthesia maintenance	10(26.3)	12(31.6)	26(68.4)
Anesthesia recovery	0(0.0)	0(0.0)	0(0.0)
PACU	0(0.0)	0(0.0)	0(0.0)
Sinus bradycardia			
Anesthesia induction	0(0.0)	0(0.0)	0(0.0)
Anesthesia maintenance	1(2.6)	1(2.6)	1(2.6)
Anesthesia recovery	0(0.0)	0(0.0)	0(0.0)
PACU	0(0.0)	0(0.0)	0(0.0)
Hypoxemia			
Anesthesia induction	0(0.0)	0(0.0)	0(0.0)
Anesthesia maintenance	0(0.0)	0(0.0)	0(0.0)
Anesthesia recovery	0(0.0)	0(0.0)	1(2.6)
PACU	0(0.0)	0(0.0)	0(0.0)
Agitation and tremors	0(0.0)	0(0.0)	0(0.0)
Injection pain	0(0.0)	2(5.3)	7(18.4)
Movement during procedure	1(2.6)	1(2.6)	1(2.6)
Intraoperative awareness	0(0.0)	0(0.0)	0(0.0)
Postoperative nausea and vomiting	0(0.0)	0(0.0)	2(5.3)
Airway intervention after LMA extraction	1(2.6)	0(0.0)	1(2.6)
Complications at POD14	0(0.0)	0(0.0)	0(0.0)

Data are presented as numbers (percentages)

Abbreviations: LMA, laryngeal mask airway; PACU, postanesthesia care unit; POD14, 14 days after surgery; RT, remimazolam tosilate

## Discussion

The present study was the first to evaluate RT in general anesthesia for day surgery. RT induction was as rapid as propofol’s. A fast and complete recovery from general anesthesia could be achieved with the use of flumazenil. Although higher BIS values were observed in patients treated with RT, no intraoperative awareness was reported. Vasopressor dosage, injection pain and serum TG levels were decreased in patients who received RT with or without flumazenil compared with propofol.

Because minimally invasive surgery is now well established, more procedures can be performed as day surgeries [1]. Advantages of IV anesthesia with a LMA, which is commonly used for the induction and maintenance of general anesthesia during day surgery, include steady induction, fast recovery and relatively simple administration [18]. To achieve enhanced recovery after a day surgery, short-acting anesthetics are required. RT has been approved for clinical use in China by the National Medical Products Administration as a novel ultrashort-acting benzodiazepine for general anesthesia and sedation since 2021. In this study, RT (0.3 mg/kg, iv) led to rapid LoC during induction in a similar manner to propofol. However, a prior work reported increased induction times with RT compared with propofol when administered as a constant infusion for procedural sedation during intestinal endoscopy [11, 12]. Similar findings were reported when remimazolam besylate was used [7], suggesting that a single IV dose of remimazolam besylate or RT permits faster anesthesia induction than a constant infusion.

The dosing we chose for RT in this study was based on the results of Phase II and III clinical trials of RT [11] and the report on remimazolam besylate for general anesthesia [7]. BIS was used in this study to monitor anesthesia depth. We found that patients who received RT had higher BIS values than those receiving propofol from the time point of reaching LoC to the discontinuation of the study drug, and more patients treated with RT had BIS values > 60 during maintenance compared with propofol, while fewer had BIS values < 40, which is consistent with the findings of previous studies [7, 19] that evaluated remimazolam besylate. However, after LMA extraction and operating room discharge, BIS values were found to be lower only in the RT group but not the RT + flumazenil group compared with the propofol group in this study, indicating that RT achieved a similar recovery from sedation as propofol only when patients were treated with flumazenil. One previous study showed that BIS values during anesthesia with remimazolam were significantly higher than those with propofol despite comparable anesthetic effects [7]. The higher BIS value is partly due to an increment of β waves during administration of benzodiazepine [20]. The validity of the BIS value in anesthesia with benzodiazepine has been controversial [21, 22]. As a result, BIS might not properly reflect the depth of anesthesia with RT compared with propofol. Other efforts have been made in monitoring the depth of remimazolam anesthesia, like Sedline [23], however, it is still not ideal and further studies are needed.

RT was found to achieve a similar recovery time from general anesthesia compared with propofol only when patients were treated with flumazenil 10 min after RT discontinuation in this study. It’s worth noting that BIS values were different between the RT combined with flumazenil group and propofol group, which may affect the result of recovery time. However, RT was reported to have a faster recovery from procedural sedation after upper gastrointestinal endoscopy compared with propofol when administered via intermittent IV injection [11]. The use of RT through continuous infusion in this study may account for the prolonged recovery time. A prior study suggested that when using a controlled infusion of remimazolam or propofol to maintain general anesthesia, the context-sensitive half-time (CSHT) of remimazolam had little relationship with the infusion time, while the CSHT of propofol increased with a prolonged infusion time [24]. Therefore, for patients undergoing day surgeries, which generally have shorter procedural times, propofol may allow for a faster recovery from general anesthesia compared with RT. In the present study, SBP, DBP and HR were higher in the RT or RT + flumazenil group compared to the propofol group at several time points from induction to operating room discharge, while lower dosing of vasopressors and fewer cases of treatment-related hypotension were observed in the patients treated with RT. This may be partly attributed to a lower rate of cardiovascular depression of RT compared with propofol, which are also consistent with previous reports [11, 12]. More patients in the propofol group were under deep anesthesia with BIS values < 40 which also needs to be taken into consideration [25]. Lighter anesthesia may be induced by RT.

A recent study reported lower physical comfort and emotional state recovery quality among patients who received remimazolam besylate keeping BIS ranges between 40 and 60 compared with propofol for urologic surgery on POD1 [8]. The cognitive evaluation performed in this study showed that RT and propofol had similar cognitive recovery during early recovery period, as shown by the MMSE score and telephone interview. Similar results of short-term recovery quality were also observed using PostopQRS in the present work. It suggested that RT and propofol may permit similar recovery quality. However, considering the higher BIS values in the patients receiving RT in our study, remimazolam besylate may have a similar recovery profile to RT under the same depth of anesthesia, which needs further study. It’s also worth pointing out that this real world study included patients aged 18–75 years old and the number of patients aged > 65 years old in each group was balanced. Similar inclusion criteria have been adopted by other studies [26, 27]. Adults aged > 65 years old are the fastest growing segment of the population around the world [28]. As the population ages, the demand for surgery is projected to increase by 18% [29]. Given the improving health status and functional independence in older individuals, it has been considered to change cut-off age from 65 to 75 years old to define “elderly” [30]. Of course, the use of RT in older patients needs further study.

Adverse events linked to flumazenil include pain on injection, agitation and tremors, flushing, dizziness, sweating or shivering, headache, blurred vision and ringing in the ears [31]. The safety of the use of flumazenil was also considered in this study. No AEs related to flumazenil were observed in patients treated with RT in conjunction with flumazenil prior to PACU discharge. Two patients in the RT + flumazenil group experienced injection pain during anesthesia induction, not anesthesia recovery. The injection pain therefore did not occur because of the flumazenil. A prior study suggested that 1.0 to 10.0 mg of flumazenil can increase the risk of AEs in patients who visited the emergency department with impaired consciousness due to benzodiazepine overdose [32]. However, the average dose of flumazenil used in this study was 0.27 mg and the maximum dose was 0.70 mg. The use of flumazenil to reverse benzodiazepine sedation was also found to be safe when used after endoscopy [33] or pediatric anesthesia [34]. Therefore, small doses of flumazenil can be used to reverse the sedative and hypnotic effects of RT and enhance recovery from anesthesia. However, the routine use of flumazenil after RT requires further study.

Prior reports suggested that propofol reduces the generation of pro-inflammatory cytokines and exerts a neuroprotective effect by maintaining Th17/Treg cell balance [2]. Upregulation of CD11 and CD18 on the surface of neutrophils is an important marker of the inflammatory response [35]. Hence, inflammatory factors and the expression of CD11b/CD18 for neutrophils and CD39/CD73 for Tregs [36] were measured in this study. No significant differences were detected in either set of surface markers between the three groups, suggesting that RT may have a similar effect on the patient’s inflammatory profile compared with propofol. A prior work suggested that propofol induces marked changes in lipid profile and a modest increase in total TG [37]. In the present study, increased TG expression after anesthesia was observed in the propofol group but not the RT groups.

This study has several limitations. First, because the present study is limited to patients who underwent day surgery with an LMA, the efficacy and safety of RT during procedures that require intubation may be different. Second, NPY was detected before PACU discharge in this study when patients might fully recover from sleep inhibition. Changes in NPY are suggested to be detected before, during and after the procedures in the future study. Third, propofol and RT may reach different levels of anesthesia depth according to the BIS values. The depth of anesthesia may affect the hemodynamic stability and the recovery time. However, BIS monitoring may not properly reflect the depth of anesthesia with remimazolam. Appropriate ranges of BIS and alternative methods to monitor the depth of RT anesthesia need more researches. Fourth, due to limited experience with the drug, the optimal induction dose and the recovery profile of RT still need to be explored. Anesthesiologists need more experience with RT to its full advantage and initiate tapering off early enough to allow for the fastest recovery. Lastly, patients over 75 years old and above ASA II were not included in this study. Hence, the efficacy and safety of RT and flumazenil in older patients and those with comorbidities requires further study. Larger trials with a more vulnerable patient population are needed before RT can be recommended for general use.

## Conclusions

This study demonstrated that RT permits rapid induction and equivalent postoperative recovery quality compared with propofol in general anesthesia for day surgery. However, there was a prolonged recovery time of RT, but not RT in conjunction with flumazenil compared with propofol. RT had a superior safety profile to propofol in terms of hypotension and injection pain. Less changes in lipid metabolism were also observed in the patients receiving RT.

## Electronic supplementary material

Below is the link to the electronic supplementary material.

Supplementary Material 1

Supplementary Material 2

## Data Availability

Individual participant data that underlies the results of this article can be accessed with approval from the corresponding author on reasonable request. The study protocol and statistical analysis plan is also available.

## Abbreviations

IV: intravenous
RT: remimazolam tosilate
ASA: anesthesiologists physical status
LMA: laryngeal mask airway
LoC: loss of consciousness
BIS: bispectral index
SBP: systolic blood pressure
HR: heart rate
SpO2: blood oxygen saturation
PACU: post-anesthesia care unit
MOAA/S: modified observer’s alertness/sedation scale
MMSE: mini-mental state examination
PostopQRS: post-operative quality of recovery scale
VAS: visual analogue scale
POD1 and POD14: 1 and 14 days after surgery
DBP: diastolic blood pressure
NPY: neuropeptide Y
IL: interleukin
TG: triglyceride
VLDL: very low density lipoprotein
AEs: adverse events
SD: standard deviation
CSHT: context-sensitive half-time
