# Supplementary material for: Efficacy and safety of remimazolam tosilate versus propofol in patients undergoing day surgery: a prospective randomized controlled trial
Source: BMC Anesthesiol. 2023 May 26;23:182. doi: 10.1186/s12871-023-02092-2 (PMC10214677; doi:10.1186/s12871-023-02092-2)
Supplement: Supplementary file 1 — Supplementary Material 1 [file 12871_2023_2092_MOESM1_ESM.docx]

**Supplementary Table 1** Changes of biomarkers of neutrophil and Treg cells in peripheral blood mononuclear cells

| Changes before and after anesthesia (%) | RT  (n = 38) | RT + flumazenil  (n = 38) | Propofol  (n = 38) | P value |
| --- | --- | --- | --- | --- |
| CD39(+) | -1.78±11.17 | -0.67±6.59 | -0.83±5.21 | 0.922 |
| CD73(+) | -2.63±10.00 | -2.09±6.82 | 1.74±4.49 | 0.444 |
| CD39(+)CD73(+) | -0.50±2.00 | -0.05±1.82 | 0.28±2.02 | 0.374 |
| CD11b(+) | -2.59±12.07 | -1.49±6.01 | 0.20±4.96 | 0.644 |
| CD18(+） | 0.34±1.97 | -5.78±22.00 | -7.30±26.56 | 0.570 |
| CD11b(+)CD18(+） | -2.63±12.21 | -8.49±22.36 | 1.93±6.60 | 0.230 |

Data are presented as mean ± SD.

**Abbreviations:** RT, remimazolam tosilate; SD, standard deviation.


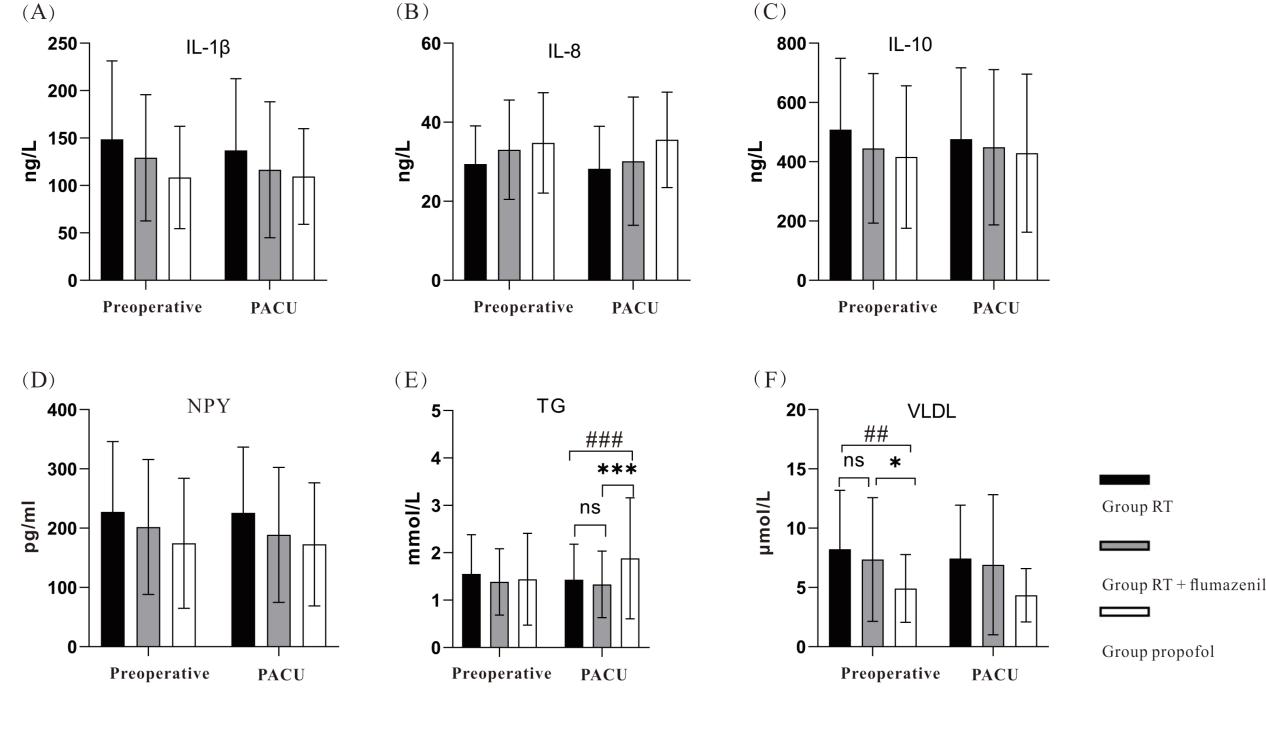


**Supplementary Fig. 1** The inflammatory factors and lipid profiles measured in blood samples before anesthesia induction and at PACU discharge. (A) Serum IL-1β levels; (B) serum IL-8 levels; (C) serum IL-10 levels; (D) serum NPY levels; (E) serum TG levels; (F) serum VLDL levels. Data are shown as mean (± standard deviation). IL, interleukin; NPY, neuropeptide Y; TG, triglyceride; VLDL, very low density lipoprotein; PACU, postanesthesia care unit; RT, remimazolam tosilate.

*P < 0.05, ***P < 0.001, RT + flumazenil versus propofol group; ##P < 0.01, ###P < 0.001, RT versus propofol group; ns: no statistic difference.
