# Supplementary material for: Efficacy and safety of remimazolam tosilate versus propofol in patients undergoing day surgery: a prospective randomized controlled trial
Source: BMC Anesthesiol. 2023 May 26;23:182. doi: 10.1186/s12871-023-02092-2 (PMC10214677; doi:10.1186/s12871-023-02092-2)
Supplement: Supplementary file 2 — Supplementary Material 2 [file 12871_2023_2092_MOESM2_ESM.docx]

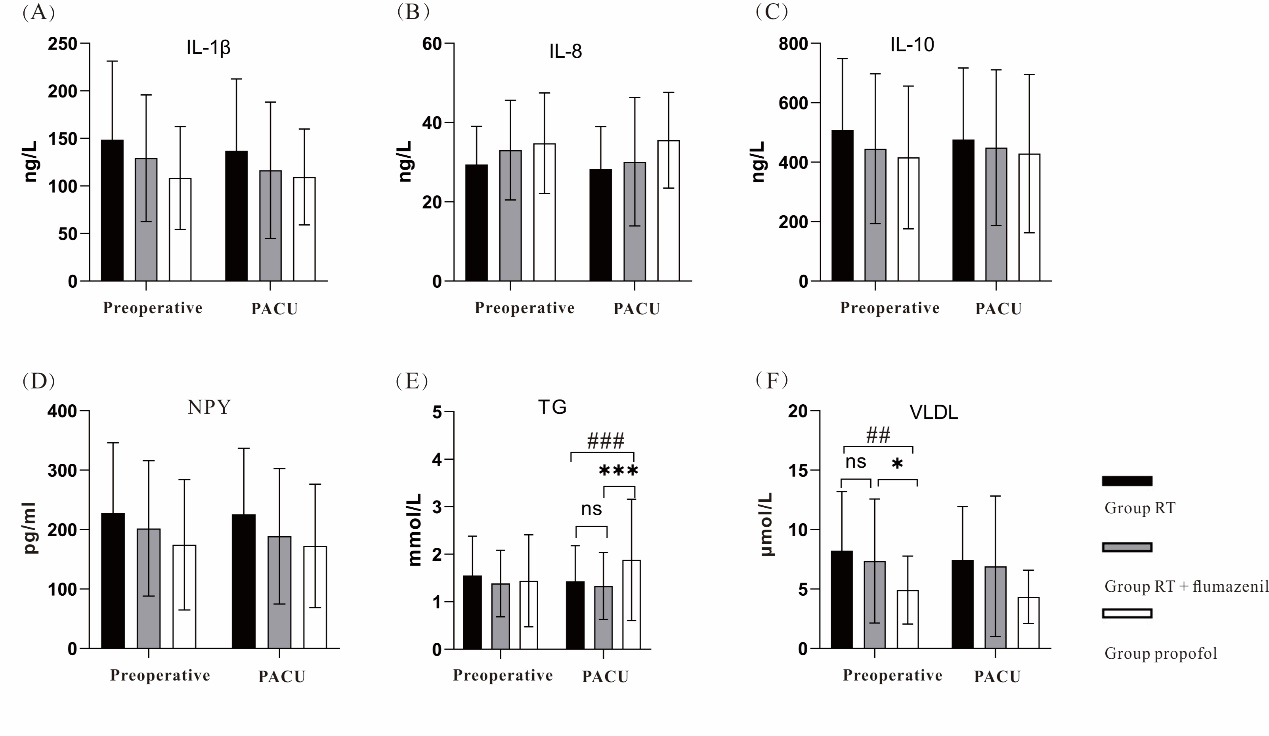


**Supplementary Fig. 1** The inflammatory factors and lipid profiles measured in blood samples before anesthesia induction and at PACU discharge. (A) Serum IL-1β levels; (B) serum IL-8 levels; (C) serum IL-10 levels; (D) serum NPY levels; (E) serum TG levels; (F) serum VLDL levels. Data are shown as mean ± SD. SD, standard deviation, IL, interleukin; NPY, neuropeptide Y; TG, triglyceride; VLDL, very low density lipoprotein; PACU, postanesthesia care unit; RT, remimazolam tosilate.

*P < 0.05, ***P < 0.001, RT + flumazenil versus propofol group; ##P < 0.01, ###P < 0.001, RT versus propofol group; ns: no statistic difference.
